# Is the difference in the volume of the pharyngeal space, as measured by acoustic pharyngometry, before and after tonsillectomy proportional to the volume of the excised tonsils?

**DOI:** 10.6061/clinics/2016(05)08

**Published:** 2016-05

**Authors:** Renata C. Di Francesco, Mariana Schmidt Kreibich

**Affiliations:** Faculdade de Medicina da Universidade de São Paulo Departamento de Otolaringologia, São Paulo/SP, Brazil

**Keywords:** Tonsil, Tonsillectomy, Obstructive Sleep Apnea, Children

## Abstract

**OBJECTIVE:**

Adenotonsillectomy is recognized as an effective therapy for snoring and sleep disorders in children. It is important to understand whether adenotonsillectomy significantly increases the volume of the pharyngeal space. The goal of this study was to evaluate the change in oropharyngeal volume after adenotonsillectomy and the correlation of this change with the objective volume of the tonsils and body mass index.

**METHODS:**

We included 27 subjects (14 males) with snoring caused by tonsil and adenoid hypertrophy. The mean age of the subjects was 7.92 (±2.52) years. Children with craniofacial malformations or neuromuscular diseases or syndromes were excluded. The parents/caregivers answered an adapted questionnaire regarding sleep-disordered breathing. All patients were subjected to weight and height measurements and body mass index was calculated. The subjects underwent pharyngometry before and after adenotonsillectomy and the volume of both excised tonsils together was measured in cm^3^ in the operating room.

**RESULTS:**

Pharyngometric analysis showed that the mean pharyngeal volume was 28.63 (±5.57) cm^3^ before surgery and 31.23 (±6.76) cm^3^ after surgery; the volume of the oropharynx was significantly increased post-surgery (*p*=0.015, Wilcoxon test). No correlation was found between the objective tonsil volume and the post-surgical volume increase (*p*=0.6885). There was a fair correlation between the oropharyngeal volume and body mass index (*p*=0.0224).

**CONCLUSION:**

Adenotonsillectomy increases the volume of the pharyngeal space, but this increase does not correlate with the objective tonsil size. Furthermore, greater BMI was associated with a smaller increase in the pharyngeal volume. Oropharyngeal structures and craniofacial morphology may also play a role in the increase in oropharyngeal volume.

## INTRODUCTION

Adenotonsillectomy (AT) is widely recognized and recommended by the American Academy of Pediatrics (AAP) as an effective first-line therapy for snoring and sleep-disordered breathing (SDB) in children 1. Snoring and SDB, which affect approximately 11% of children aged 2-8 years old, are anatomically caused by the enlargement of the tonsils and adenoids. Primary snoring itself, even in the absence of sleep apnea, has been significantly associated with behavioral difficulties and neurocognitive impairment in pediatric patients 2.

Objective published data have shown that the quantity of snoring decreases after AT 3; however, objective measures of tonsil size (weight and volume) were not directly correlated with objective snoring measures 2. The assessment of tonsillar hypertrophy is frequently limited to direct visualization through the mouth 4 and the clinical utility of tonsil size as a predictor of SDB presence or severity is unclear, despite the common use of this technique 5.

The persistence of symptoms after AT is common, particularly in obese children, as symptom persistence is observed in 50% of these children 6,7. Therefore, it is important to understand whether there is an actual increase in the volume of the pharyngeal space after AT.

We used acoustic pharyngometry to measure pharyngeal volume because it is a non-invasive technique that has been used to assess the pharyngeal size in snorers 8, including children 4.

The goal of this study was to evaluate the change in oropharyngeal volume after AT and the correlations of this change with the objective volume of the tonsils and body mass index (BMI).

## METHODS

This study was approved by the Hospital das Clínicas Committee on Ethics before patient enrolment was initiated. The parents and caregivers of the patients signed informed consent forms.

**Figure 1:**
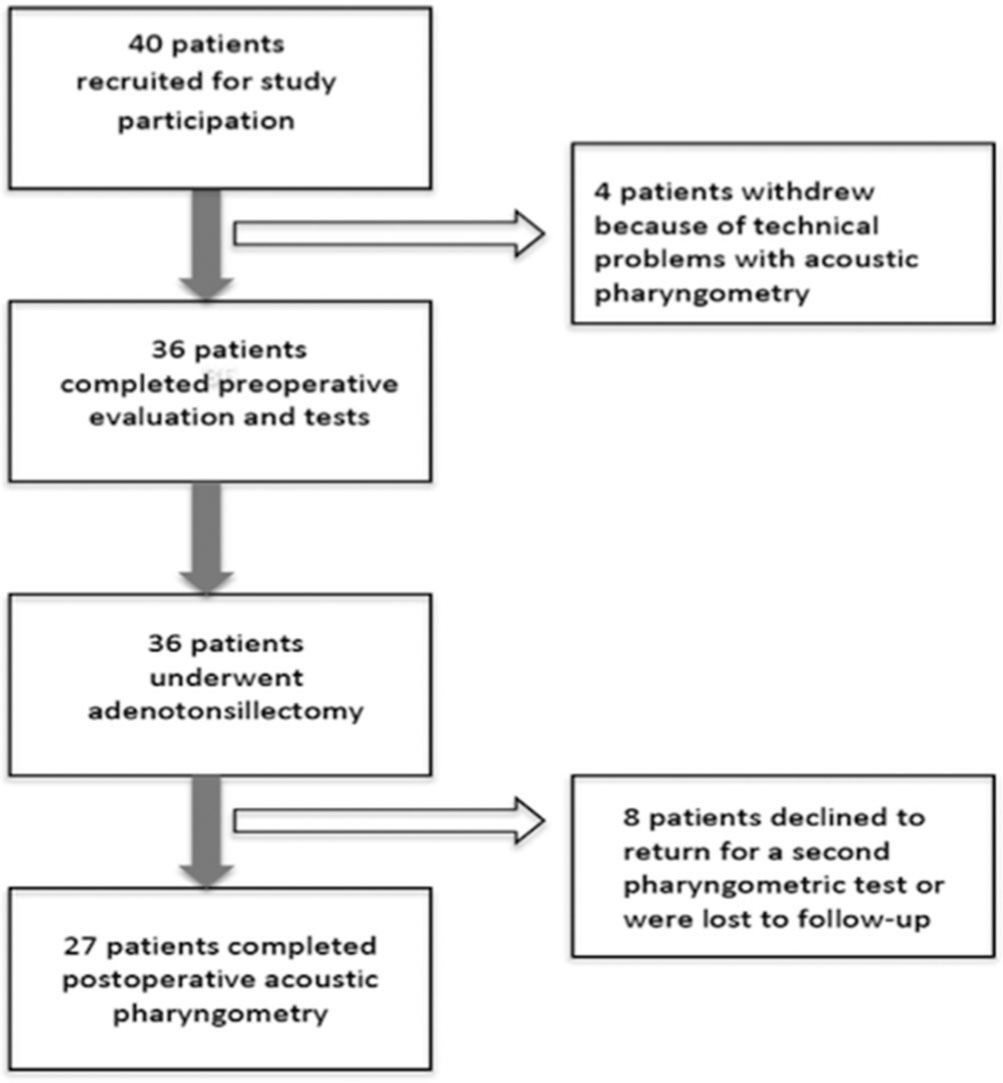
Patient selection.

We selected forty children and teenagers aged from 4 to 14 years old with snoring and mouth breathing caused by tonsil and adenoid hypertrophy of grades 3+ and 4+ who presented at the Department of Otolaryngology of the University of São Paulo Medical School for AT. Of these subjects, 27 were included in the current study (Figure 1).

The exclusion criteria consisted of the following: craniofacial malformations and neuromuscular diseases or syndromes. The parents and caregivers answered an adapted questionnaire on SDB developed by Goodwin et al., 2004 9 that was translated into Portuguese by Petry et al. in 2008 10. The responses to the questions consisted of “yes” or "no" answers (Chart 1).

All of the patients underwent measurements of weight and height. BMI and corresponding z-scores were calculated for each patient and compared using BMI z-scores derived from the 2000 Centers for Disease Control and Prevention growth charts 11.

The airway was systematically evaluated. The physical examination was performed using a standardized approach. Tonsil size was evaluated pre-operatively in the office setting using the Brodsky grading scale 12.

Pharyngometric data were collected for all of the subjects. The subjects sat in an upright position on a straight-back chair and breathed orally through an acoustic pharyngometer (Eccovision® Pharyngometer, Sleep Group Solutions, Miami, FL, USA) with the aid of a nose clip. A disposable mouthpiece was used to stabilize the tongue and to provide a reproducible bite position. At a normal resting lung volume (functional residual capacity), the subjects were instructed to pause breathing at end-exhalation while maintaining a relaxed airway, and during this pause, the acoustic measurement of the upper airway was performed. The measurements were repeated three times. The data were plotted as the product of the cross-sectional area *versus* the distance from the incisors. The oropharyngeal volume was calculated as the product of the cross-sectional area of the oropharyngeal junction up to but excluding the glottis.

All patients underwent AT. The volume of both excised tonsils together was measured in cubic centimeters based on the volumetric displacement of saline solution in a burette in the operating room.

Pharyngometry was repeated one month after the surgery. The pre- and post-surgery volumes of the oropharynx were compared and the difference in the oropharyngeal volume was compared with the volume of the tonsils.

### Statistics

The sample size was calculated to reach a default power of 0.80, and 15 subjects were needed. We included 27 subjects and the power obtained was 0.96. Statistical analysis was performed using STATA version 8.2 software (StataCorp LP, College Station, TX, USA). The McNemer test was used to evaluate the evolution of symptoms after surgery. The data are presented as the means and standard deviation. Wilcoxon sign tests were used to compare the means and Pearson's correlation coefficients were applied to compare the continuous variables.

## RESULTS

### Patient characteristics

Twenty-seven (14 males) of the 40 children who underwent AT during the study period were included in the study. The mean age was 7.92 (±2.52) years and detailed demographic information is presented in Table 1.

All patients presented with various symptoms of SDB and 96% of patients presented with snoring (Table 2).

### Comparison of symptoms between before and after surgery

Table 2 shows that there was an improvement in the majority of symptoms after surgery.

### Comparative analysis of the pharyngometry results between pre-treatment and post-treatment

**Figure 2:**
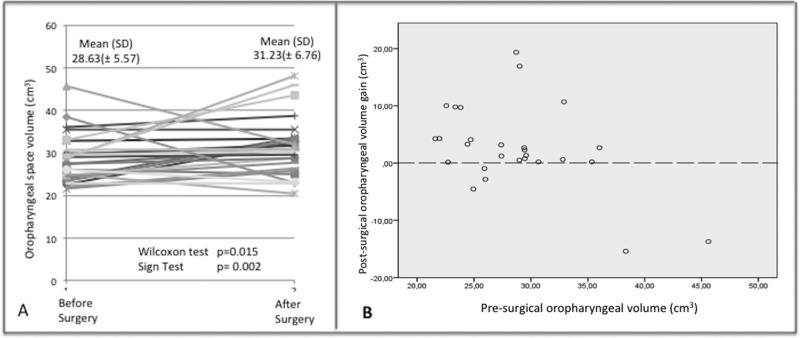
A Comparison of pharyngeal space volume between before and after surgery. B Pre-surgical pharyngeal volume and increase in the pharyngeal volume.

Pharyngometric analysis showed that there was a significant increase in the oropharyngeal volume after surgery (Figure 2 A).

Figure 2 B shows line plots comparing each of the airway volume measurements before surgery and the volume increase after surgery in each subject. These results show that some subjects exhibited negative changes in volume, mainly for oral cavity volume and total airway volume. Interestingly, the subjects with negative volume changes exhibited larger pre-surgical airway volumes.

**Figure 3:**
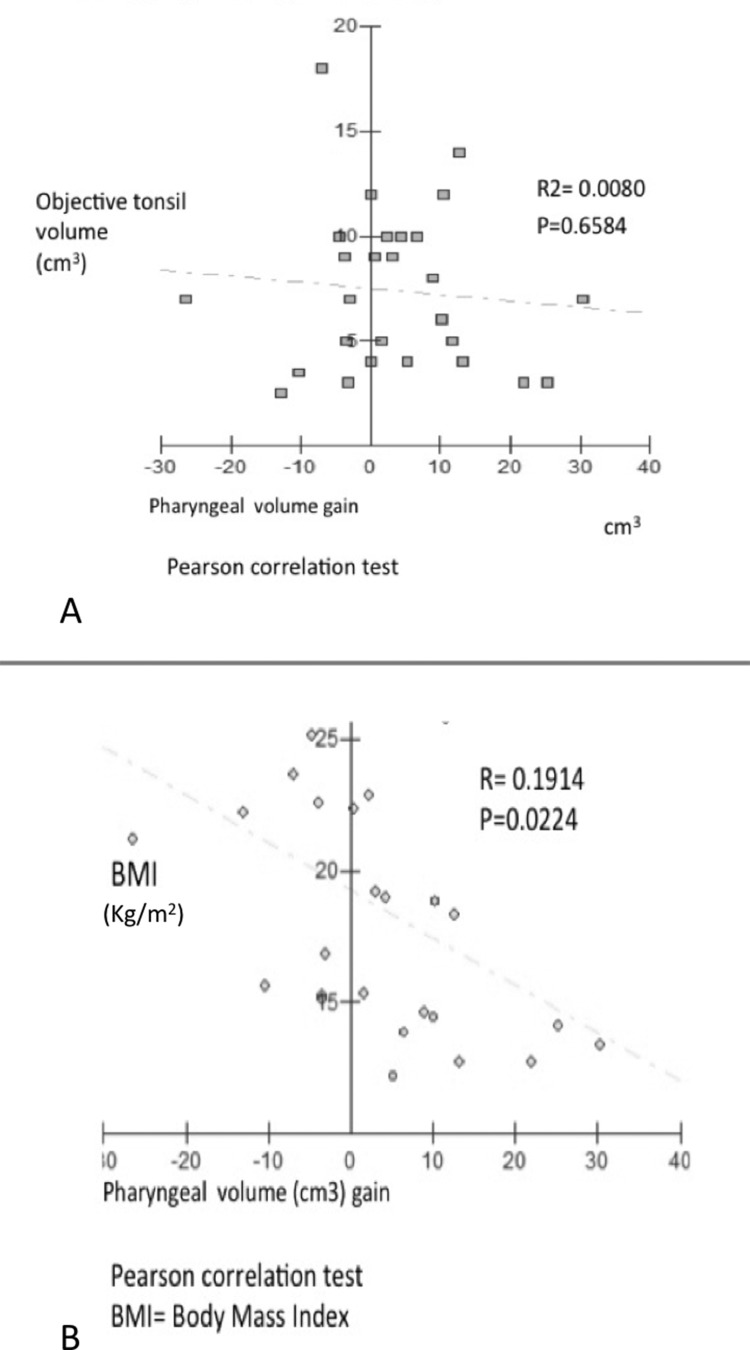
A Correlation of the increase in pharyngeal volume with tonsil volume. B Correlation of the increase in pharyngeal volume with body mass index.

No correlation was found between the objective tonsil volume and the post-treatment increase in Pharyngeal volume (*p*=0.6885) (Figure 3 A).

### Post-treatment increase in oropharyngeal volume and BMI

The correlation between the increase in oropharyngeal volume and BMI was fair. This result suggests that higher BMI was correlated with a smaller pharyngeal volume post-surgery (Figure 3 B).

## DISCUSSION

This study found that tonsillectomy leads to an increase in oropharyngeal volume. This finding should be expected because this procedure reduced the mass of the pharynx. Surprisingly, when analyzing the increase in pharyngeal volume after tonsillectomy case by case, variations in this volume increase were observed. In most cases, increased pharyngeal volume was observed. However, some patients did not show a difference in volume and the two patients with the largest pre-surgical pharyngeal volumes showed a decreased pharyngeal volume after surgery.

The intention of this study was to evaluate the pharyngeal space. Therefore, we utilized acoustic pharyngometry because it represents a simple, quick, non-invasive method for measuring the dimensions of the upper airway. Previous authors have demonstrated that children are capable of adequately undergoing this procedure; 80% of 4-year-old children were able to undergo the measurement satisfactorily 4, as were 78% of children aged 8–11 years 8. In clinical practice, tomographic and radiographic methods (computed tomography (CT) scans of the pharynx) are limited to diagnostic use in complex cases because of the cost and the higher level of radiation exposure 4. Alternatives such as magnetic resonance imaging (MRI) or endoscopy, although considerably more accurate and comprehensive, are also much more expensive 8 and may require sedation in children. Radiographic comparisons have shown that pharyngometry enables accurate reconstructions of the geometry of other airways 13. Acoustic pharyngometry presents acceptable reproducibility. Measurements of a given subject vary by 10%, and this variation may be related to physiological changes or artifacts caused by noise or inconsistent breathing 13. This method can accurately predict SDB as diagnosed by polysomnography with a high sensitivity (90.9%) and specificity (88.4%) 14.

AT is the first-line treatment for snoring and SDB in children, but the mechanism by which tonsillectomy resolves respiratory problems in children is not well established; we believe this mechanism increased the volume of pharyngeal space. Our findings showed that patient-reported symptoms improved after surgery. Britzke et al. 2015 2 demonstrated that the post-operative improvement in snoring measures significantly but indirectly correlated with the volume of the removed tonsil tissue; their result suggested that tonsil hypertrophy indeed influenced objective snoring.

We did not perform polysomnography in this study because these examinations are costly and time-consuming and because the main goal of this study was to evaluate the pharyngeal space. Pediatric sleep questionnaires have been established to be superior to polysomnography in showing improvements in symptoms after AT 15.

We included children with snoring resulting from grades 3 and 4 adenoid and tonsil hypertrophy on the Brodsky scale because the size of the tonsil should be considered when determining the need for surgical treatment 16. The subjective tonsil size is known to be very weakly (at best) associated with the severity of SDB. Therefore, we chose to measure the objective volume of the tonsils. Although this measure was associated with the pharyngeal volume, it was not correlated with the increase in pharyngeal volume, in contrast to our expectation. Our results demonstrated that the increase in pharyngeal volume was not proportional to the tonsil size and one possible explanation for this finding may be the presence of obesity. Indeed, our results demonstrated an indirect correlation between the pharyngeal volume and BMI: the lower the BMI, the greater the increase in pharyngeal volume. This finding could be explained by the presence of adipose tissue deposits near the pharynx and neck, which generally contribute to obstructive sleep syndromes in obese children 17, as well as by increased circulating levels of inflammatory mediators 18. Although the observed correlation was only fair, this finding is reasonable given the small sample size and the existence of several other factors that may be involved in determining airway width and collapse. Airway collapse occurs as a result of the combined effects of internal airway pressure, passive tissue compliance and airway muscle activation 19.

Computational fluid dynamic end points have been determined for the assessment of AT outcomes in obese children with obstructive sleep apnea syndrome, and the airway dimensions are known to be influenced by adipose tissue 19. Ulualp and Szmuk reported that children with 3+ and 4+ grade tonsils were more likely to experience lateral wall collapse than children with 1+ and 2+ grade tonsils 20. The combinations of abnormalities in soft tissue mass and adiposity, facial anatomy and neuromuscular function might also play a role in snoring and SDB 21. The role of the tongue in determining the pharyngeal area is uncertain 22; however, after tonsillectomy, the dorsum of the tongue must adapt its position and might be displaced to the previous site of the tonsil. Such a displacement would explain why increase in pharyngeal volume may not occur in all patients and why craniofacial morphology may also be involved.

Several limitations of this study should be recognized. First, there might be some concern that the study population was biased because we included only patients with tonsil grades 3+ and 4+ and because the pharyngeal measurements were collected (by necessity) while the children were awake in a sitting position, which might not reflect the actual dimensions that are relevant to snoring in a sleeping child 2. Further studies including the mouth structures and craniofacial morphology as factors should be developed.

We conclude that AT increases the volume of the pharyngeal space. However, this increase does not correlate with the objective tonsil size and the presence of obesity may explain this lack of a correlation.

## AUTHOR CONTRIBUTIONS

Both authors made substantial contributions to the drafting, conception and design of the study; acquisition, analysis and interpretation of data for the study, and the revision and final approval of the manuscript for publication.

## Figures and Tables

**Table 1:** Demographic characteristics.

Subjects	27
Sex	14 (51.8%) male
Age (years)	7.92 (±2.52)
Weight (kilograms)	34.89±14.89
Height (meters)	1.34±0.15
BMI (kg/m^2^), z-score	18.63±14.92, 0.09±1.88
Tonsil size	Grades 3 and 4
Tonsil volume, mean (cm^3^) ± standard deviation	7.41±3.77

**Table 2:** Comparison of symptoms before and after surgery.

	Before surgery		After surgery		*p*
	yes	%	yes	%	
Does your child ever stop breathing during sleep?	11	40.74	0	0	0.001
Does your child struggle to breathe during sleep?	21	77.78	2	7.41	<0.001
Do you ever shake your child during sleep to make him/her breathe again?	10	37.04	0	0	0.002
Do your child’s lips ever turn blue or purple while he/she is sleeping?	3	11.11	0	0	0.250
Are you concerned about your child’s breathing during sleep?	19	70.3	0	0	<0.001
How often does your child snore loudly?	26	96.3	0	0	<0.001
Does your child complain of morning headaches?	12	44.44	4	14.81	0.008
Does your child fall asleep?	14	51.85	3	11.11	0.001
